# *Funneliformis mosseae* enhances drought tolerance in maize inbred lines through root transcriptomic reprogramming

**DOI:** 10.3389/fpls.2026.1808527

**Published:** 2026-06-18

**Authors:** Eszter Virág, Zoltán Zombori, Miklós Hóvári, Géza Hegedűs, László Sass, Györgyi Ferenc, Dénes Dudits, Katalin Posta

**Affiliations:** 1 Research Institute for Medicinal Plants and Herbs LTD., Budakalász, Hungary; 2 Institute of Plant Biology, HUN-REN Biological Research Centre, Szeged, Hungary; 3 Department of Information Technology and Its Applications, Faculty of Information Technology, University of Pannonia, Zalaegerszeg, Hungary; 4 Department of Microbiology and Applied Biotechnology, Institute of Genetics and Biotechnology, Hungarian University of Agriculture and Life Sciences, Gödöllő, Hungary

**Keywords:** AMF, arbuscular mycorrhizal fungi, drought stress, *Funneliformis mosseae*, genotype-dependent response, maize (Zea mays L.)

## Abstract

Drought is a major constraint on maize productivity, and its increasing frequency due to climate change necessitates improved stress adaptation strategies. Arbuscular mycorrhizal fungi (AMF) can enhance plant drought tolerance; however, the integrated mechanisms linking root development, host transcriptional regulation, and microbiome activity remain poorly understood. Here, we investigated these interactions in maize using an integrated phenotyping–transcriptomic–metatranscriptomic approach under controlled greenhouse conditions. Two inbred lines with contrasting drought tolerance (K1, tolerant; K2, sensitive) and their hybrid (KH) were grown under well-watered (60% soil moisture) and drought (30%) conditions, with or without *Funneliformis mosseae* inoculation. Mycorrhizal colonization reached 51.3–62.5% under drought, confirming effective symbiosis. RNA-seq analysis (FDR ≤ 0.05, |log_2_;FC| ≥ 1) revealed strong genotype-dependent transcriptional responses, with the drought-sensitive genotype showing the largest number of differentially expressed genes. Principal component analysis identified genotype as the primary driver of variation (PC1: 13%), followed by mycorrhizal status (PC2: 8%). AMF induced distinct, genotype-specific functional reprogramming. The drought-tolerant genotype showed moderated stress responses and maintained metabolic activity, whereas the drought-sensitive genotype exhibited sustained stress signaling and compensatory metabolic activation. The hybrid displayed a non-additive response associated with enhanced root remodeling and symbiosis-related functions. Metatranscriptomic analysis of the non-host root-associated transcript pool further revealed genotype-specific microbial functional activity patterns, ranging from activation to repression. These results demonstrate that AMF-mediated drought tolerance emerges from coordinated, genotype-dependent interactions among root development, host regulatory networks, and microbiome activity. This study provides a holobiont-level framework for understanding crop stress adaptation.

## 1 Introduction

Drought stress is one of the most critical abiotic constraints limiting maize (Zea mays L.) productivity, as it restricts water availability, disrupts nutrient uptake, and impairs plant growth under water-limited conditions. The increasing frequency and severity of drought events due to climate change further exacerbate these effects. Arbuscular mycorrhizal fungi (AMF) form symbiotic associations with most terrestrial plants and can enhance drought tolerance by improving water and nutrient acquisition, modulating hormonal balance, and alleviating oxidative stress (Guo et al., 2024; Batool et al., 2025; Li et al., 2025). Despite extensive physiological and molecular evidence, the underlying mechanisms—particularly at the level of root transcriptional regulation—remain incompletely understood.

Transcriptomic approaches such as RNA-seq have provided important insights into drought- and symbiosis-related regulatory networks (Gutjahr and Parniske, 2013). However, most previous studies have investigated either plant drought responses in non-mycorrhizal systems or AMF effects under non-stress conditions, leaving a critical gap at the intersection of drought stress and symbiotic interaction. Furthermore, although genotype-dependent variation in AMF-mediated responses has been reported (Quiroga et al., 2017; Begum et al., 2019b; Hu et al., 2020; Chang and Lin, 2025), plant responses are often treated as uniform, and the regulatory mechanisms underlying genotype-specific differences remain poorly resolved.

In our previous work, we demonstrated that *F. mosseae* colonization modulates drought adaptation in a strongly genotype-dependent manner by integrating phenomic traits with transcriptomic regulation of photosynthesis, hormone signaling, and developmental processes. *F. mosseae* stabilized photosynthetic gene expression and stress-adaptive regulation in the K1: drought-tolerant genotype, promoted root-associated developmental and hormonal reprogramming in the K2: drought-sensitive genotype, and supported heterosis-related biomass stability and flowering consistency in the KH: K1 x K2 hybrid (Virág et al., 2025).

In parallel, increasing evidence indicates that drought responses extend beyond the plant to include coordinated changes in the activity of the root-associated microbiome (Naylor and Coleman-Derr, 2018). AMF colonization can stabilize rhizosphere microbial communities and modulate host gene expression under water deficit (Chen et al., 2025), while metatranscriptomic studies demonstrate that microbial functional activity is dynamically reprogrammed under drought conditions (Tartaglia et al., 2023). However, these host and microbial responses are typically analyzed separately, limiting our understanding of how plant genotype, symbiosis, and microbiome function are integrated. Thus, a key unresolved question is whether AMF-mediated drought tolerance represents a general protective mechanism or emerges from genotype-specific coordination between root development, host transcriptional regulation, and microbiome functional activity.

To address this gap, we applied an integrated systems-level approach combining root phenotyping, transcriptomics, and function-centered metatranscriptomics. We hypothesized that: (i) AMF colonization induces distinct, genotype-dependent transcriptional programs in maize roots under drought stress; (ii) drought tolerance is associated with attenuation of stress signaling and maintenance of metabolic activity, whereas drought sensitivity involves compensatory activation of stress- and metabolism-related pathways; (iii) the functional activity of the root-associated microbiome reflects and reinforces host genotype-specific transcriptional states, resulting in coordinated holobiont-level responses. We tested these hypotheses using two maize inbred lines with contrasting drought tolerance and their hybrid under controlled water regimes, with and without *F. mosseae* inoculation. By integrating host transcriptomic and root-associated metatranscriptomic data, we aimed to identify regulatory modules linking root development, metabolic reprogramming, and microbiome-associated functional responses in a genotype-dependent manner.

The novelty of this study lies in the integration of root developmental dynamics, host transcriptional regulation, and microbiome functional activity within a unified holobiont framework, enabling the identification of coordinated, genotype-specific drought adaptation strategies rather than isolated plant or microbial responses. At the same time, this study focuses on functional transcriptional activity rather than taxonomic composition and is based on a limited number of genotypes under controlled conditions, which may constrain the generalizability of the findings.

## 2 Materials and methods

### 2.1 Experimental design

The experimental design was established based on our previously published study, ensuring methodological continuity for genotype-specific analysis of AMF-mediated drought responses in maize (Virág et al., 2025). A fully factorial experiment was established to investigate how *F. mosseae* inoculations modulates maize root responses to drought in a genotype-dependent manner. Three maize genotypes were examined: K1 (drought-tolerant parental inbred line), K2 (drought-sensitive parental inbred line), and their hybrid KH (K1 × K2). Plants were grown under two water regimes defined by soil moisture content (MC): 60% MC (well-watered control) and 30% MC (drought). For each genotype and water regime, plants were cultivated either without (M^-^) or inoculated with *F. mosseae* (M^+^), resulting in 12 treatment combinations (3 genotypes × 2 water regimes × 2 mycorrhiza statuses). A multifactorial statistical framework (genotype × treatment × AMF interaction) was applied. Root samples were collected with three biological replicates per treatment combination. Biological replication consisted of three independent samples per treatment combination, which is consistent with widely applied RNA-seq experimental designs under controlled conditions. To mitigate potential limitations in statistical power, differential expression analysis was performed using the DESeq2 framework, which applies shrinkage estimation of dispersion and fold changes to improve robustness with limited replication. In addition, downstream analyses focused on reproducible, pathway-level patterns (e.g., GSEA) rather than solely on individual gene-level changes.

To isolate the effect of mycorrhizal colonization at the primary site of symbiosis, the main analytical strategy relied on pairwise comparisons of M^+^ vs. M^-^ within each genotype and water regime. Specifically, AMF effects under control conditions were evaluated by comparing K1MC vs. K1C, K2MC vs. K2C, and KHMC vs. KHC, while AMF effects under drought were assessed by comparing K1MD vs. K1D, K2MD vs. K2D, and KHMD vs. KHD. This design enables the identification of genotype-specific and water-dependent transcriptional programs associated with mycorrhiza-mediated drought adaptation in roots.

In addition to host root transcriptomics, we performed metatranscriptomic profiling of the root-associated compartment (rhizosphere-root-environment samples corresponding to each treatment) to capture the functional activity of the non-host root-associated microbial community surrounding the root system. Integrating root RNA-seq with rhizosphere metatranscriptomes allowed us to assess whether genotype-dependent mycorrhizal effects under drought are accompanied by coordinated shifts in microbial functional gene expression, thereby providing a holobiont-level view of plant–AMF–microbiome interactions under water deficit.

Metatranscriptomic profiling was intentionally restricted to drought-treated samples in order to focus on mycorrhiza-associated microbial functional responses under stress conditions, which represented the primary biological context of this study. Consequently, constitutive microbial transcriptional states under well-watered conditions were not assessed, and interpretations are limited to drought-associated functional responses.

### 2.2 Plant material and growth conditions

Plants were grown under controlled greenhouse conditions as detailed in Virág et al. (2025). Briefly, plants of two inbred maize lines and their hybrid were grown in radio-tagged Plexiglass columns surrounded by polyvinyl chloride tubing filled with sterilized peat soil-sand mixture up to the capacity of cca. 75%, then the AMF-containing culture was mixed into the last quartile to the AMF-treated columns, while the non-treated ones were filled up with the sterilized soil mixture. The columns were placed in random order within an automatic modular phenotyping system (Photon System Instruments, Drasov, Czech Republic) in the greenhouse of the HUN-REN Biological Research Centre, Szeged, Hungary (46°14’44.0”N 20°09’54.8”E). The plants were irrigated to maintain 60% moisture content (MC) for control conditions and 30% MC for drought stress.

### 2.3 Digital imaging of the development of the root system

Root density was estimated weekly using digital imaging. Plexiglass columns were placed in a custom chamber illuminated by LED panels and photographed from four lateral perspectives and the bottom using two Canon EOS 600D cameras. To quantify the root area visible at the column-soil interface, images were processed using proprietary in-house software. This software utilized a background subtraction algorithm to isolate white root-related pixels from the dark soil matrix.

### 2.4 Measurement of root colonization

Root samples were randomly collected, thoroughly washed with tap water, and cut into 1 cm segments. The roots were cleared by immersion in 10% KOH (w/v) and incubated in a water bath at 90 °C for 60 minutes. Following this, the KOH solution was decanted, roots were rinsed with running water, then immersed in 5% acetic acid for 1–2 minutes, and stained with Pelikan-ink according to Vierheilig et al. (1998). AMF colonization was assessed using the gridline intersection method. Internal fungal structures (hyphae, arbuscules) were examined under a stereomicroscope at 100× magnification, and the percentage of root colonization was calculated according to Giovannetti and Mosse (1980) (Giovannetti and Mosse, 1980). The presence or absence of AMF is recorded for approximately 100 intersections of the gridlines with the roots.

### 2.5 RNA isolation and transcriptomic analysis of plant roots

Root transcriptomic profiling was conducted following a methodology consistent with our previous study (Virág et al., 2025). In brief, the samples for the RNA isolation were collected from roots of maize plants at an identical part of the root system, and frozen immediately in liquid nitrogen. The samples were ground in a mortar into fine powder, and approx 50-75 µg of this material was used for total RNA isolation. The procedure was carried out using E.Z.N.A.^®^ Fungal RNA Mini Kit (Omega Bio-tek Inc., Norcross, GA, USA) following the manufacturer’s instructions. RNA integrity number, RIN ≥ 7 was determined before sequencing. Sequencing libraries were prepared using Illumina TruSeq kits and sequenced on an Illumina platform to generate paired-end reads.

Raw sequencing data quality was assessed using FastQC v0.12.1, and adapter trimming and quality filtering were performed using Trimmomatic v0.39. Only high-quality reads (Phred score ≥ 30) were retained for downstream analyses. On average, 23–27 million paired-end reads were obtained per sample. Filtered reads were mapped to the *Zea mays* reference transcriptome (NCBI GCF_902167145.1, RNA.fasta) to the transcript abundance estimation after that transcript quantification was performed at the transcript level based on mapped reads. Mapping was performed using a splice-aware aligner, resulting in an average mapping efficiency of 82–91%. Only uniquely mapped reads were used for transcript quantification. Transcript abundance was estimated at the transcript level and summarized to gene-level counts for differential expression analysis. Sample quality and consistency were further evaluated using principal component analysis (PCA) and sample distance clustering.

The differential expression analysis was carried out using DESeq2, a Bioconductor package (version 3.20). Differentially expressed genes (DEGs) between mycorrhizal (M^+^) and non-mycorrhizal (M^-^) root samples were identified via pairwise comparisons within each genotype and water regime, with significance thresholds of false discovery rate (FDR) ≤ 0.05 and (log_2_; fold change) ≥ 1.

Functional annotation of DEGs was performed using the Functional Analysis module in OmicsBox v.3.4.6 (Blast2GO-based) (Götz et al., 2008), assigning Gene Ontology (GO) terms and pathway terms to facilitate biological interpretation.

Individual genes from key enriched pathways related to ABA and JA signaling, root hair development, AM-associated processes were selected for RPM-based expression profiling to identify regulatory patterns underlying genotype-dependent stress responses and symbiotic interactions.

### 2.6 Gene set enrichment and pathway analysis of root transcriptomes

GSEA was performed according to Subramanian et al. (2005) (Subramanian et al., 2005) on root transcriptomic data to identify coordinated pathway-level responses associated with *F. mosseae* colonization under drought stress.

Ranked gene lists were generated from pairwise differential expression analyses comparing mycorrhizal (M^+^) and non-mycorrhizal (M^-^) root samples within each genotype under drought conditions (K1MD vs. K1D, K2MD vs. K2D, and KHMD vs. KHD). Genes were ranked based on the signed Wald statistic obtained from DESeq2, which integrates both the magnitude and direction of differential expression. Positive values correspond to genes up-regulated in mycorrhizal samples, whereas negative values correspond to genes down-regulated relative to non-mycorrhizal controls.

Gene sets were defined based on curated pathway annotations from the Plant Reactome database. To ensure statistical robustness and biological interpretability, gene sets containing fewer than 10 or more than 500 genes were excluded.

Enrichment scores were calculated using a weighted enrichment statistic, and statistical significance was assessed using 1,000 permutations. Normalized enrichment scores (NES) were computed to allow comparison across gene sets, and multiple testing correction was performed using the Benjamini–Hochberg method (FDR ≤ 0.05).

Gene set enrichment results were further integrated with functional annotation analyses performed in OmicsBox (BioBam Bioinformatics S.L., Valencia, Spain; version 3.4.6) to support biological interpretation at Gene Ontology and pathway levels.

This GSEA-based approach enables the detection of subtle but coordinated transcriptional shifts at the pathway level, providing mechanistic insight into mycorrhiza-mediated modulation of root stress responses beyond individual differentially expressed genes.

### 2.7 Metatranscriptomic analysis, host-depleted transcriptome-based holobiont functional profiling

Metatranscriptomic profiling of the root-associated compartment was conducted using a host-depletion strategy followed by *de novo* assembly and functional annotation workflows. Raw paired-end reads were quality-filtered and adapter-trimmed using the Transcriptomics module. To remove host-derived sequences, filtered reads were mapped against the Zea mays reference transcriptome (NCBI GCF_902167145.1, RNA.fasta). Reads that did not align to the maize transcriptome were exported as unmapped reads and retained for downstream analyses as non-host root-associated microbial reads.

Host-depleted reads from drought-treated samples were combined for co-assembly and assembled *de novo* into transcript contigs using Trinity v2.13.2 (Grabherr et al., 2011; Haas et al., 2013). Co-assembly was employed to generate a unified reference for downstream quantification and to improve assembly continuity across samples. Host-depleted reads from each sample were then mapped back to the assembled contigs to generate contig-level count matrices.

The minimum contig length threshold of 200 bp was selected to balance assembly sensitivity with functional annotation reliability in the context of short-read metatranscriptomic data. Given the applied paired-end read length, contigs shorter than 200 bp frequently lack sufficient coding sequence information to support confident homology-based annotation, leading to ambiguous or domain-level assignments. Implementing a 200 bp cutoff reduces spurious annotations while retaining a substantial proportion of biologically meaningful microbial transcripts, a practice commonly adopted in metatranscriptomic assemblies to improve annotation robustness. Reads showing partial or ambiguous alignment to both host and microbial references were handled conservatively during the host-depletion step. Specifically, reads that mapped to the maize reference transcriptome under the applied alignment criteria were classified as host-derived and excluded from downstream metatranscriptomic analyses. Only reads failing to align to the host reference were retained for *de novo* assembly and microbial functional profiling, minimizing the risk of host sequence carryover in the assembled metatranscriptome.

Functional annotation of assembled contigs was performed using the Functional Analysis module in OmicsBox v.3.4.6 (Blast2GO-based) (Götz et al., 2008) integrating BLAST/DIAMOND-based similarity searches against the NCBI non-redundant (nr) protein database and UniProtKB databases.

Contigs were translated into putative coding sequences prior to annotation, and similarity searches were conducted using DIAMOND with default parameters. Hits were filtered based on standard criteria, including minimum e-value thresholds (e.g., ≤ 1e−5), minimum alignment length, and sequence coverage to ensure reliable functional assignment. To minimize spurious or domain-only assignments common in complex microbial datasets, alignments were filtered based on minimum high-scoring segment pair (HSP) length and HSP–hit coverage thresholds prior to GeneOntology (GO) and pathway mapping. GO terms and pathway annotations were assigned using Blast2GO-based mapping, and functional classification was further supported by pathway-level annotations where available. This pipeline enables function-centered interpretation of metatranscriptomic activity while minimizing spurious or low-confidence annotations.

Differential abundance analyses were conducted as within-genotype pairwise comparisons of mycorrhizal (M^+^) versus non-mycorrhizal (M^-^) samples under drought stress. Count data were normalized using TMM normalization, and low-abundance contigs were filtered based on counts per million (CPM) thresholds requiring detection in at least two samples. Functional enrichment analyses were subsequently performed on differentially abundant contigs to identify mycorrhiza-associated shifts in rhizosphere microbial activity under drought conditions.

The metatranscriptomic analysis was intentionally designed to resolve functional metabolic activity rather than taxonomic composition, as transcriptional functional states provide a more direct representation of the active biochemical processes shaping root-holobiont drought responses. Because multiple microbial taxa can perform overlapping metabolic functions, a function-centered framework enables biologically meaningful interpretation of holobiont-level stress adaptation independent of community compositional variation.

Because no explicit taxonomic assignment was performed, the detected transcripts cannot be unambiguously attributed specifically to *F. mosseae*, but instead represent the active non-host root-associated microbial transcript pool.

### 2.8 Statistical analysis and reproducibility

All statistical analyses were performed in R using established Bioconductor workflows. Statistical significance was set at p <0.05 by root colonization and data are presented as mean ± standard deviation (SD). Differential expression analysis relied on the DESeq2 framework, including gene-wise dispersion estimation with empirical Bayes shrinkage and hypothesis testing using the Wald test, followed by Benjamini–Hochberg false discovery rate (FDR) correction. Unless otherwise stated, genes with adjusted p-value ≤ 0.05 and |log_2_; fold change| ≥ 1 were considered significant. Biological replication consisted of three independent samples per treatment combination, and all downstream analyses were conducted on replicate-resolved count matrices. To assess potential batch or technical effects, exploratory data analyses including principal component analysis (PCA) and sample distance clustering were performed prior to differential testing. No systematic batch structure requiring correction was detected. For enrichment analyses, significance thresholds were based on multiple-testing–adjusted FDR values, and only gene sets passing the predefined adjusted significance cutoff were interpreted. All reported statistical tests were two-sided, and default parameters were used unless explicitly specified. Software package versions and computational settings are provided to ensure full analytical reproducibility.

Effect sizes were interpreted based on log_2_; fold change values for gene-level analyses and normalized enrichment scores (NES) for pathway-level analyses.

The observed clustering patterns were consistent across biological replicates, supporting the robustness of genotype- and treatment-associated variation.

## 3 Results

### 3.1 Growth kinetics and AMF colonization under drought stress

Detailed quantitative analysis and statistical evaluation of root growth kinetics and architectural traits in the same experimental system were previously reported by Virág et al. (2025). In the present study, root growth observations are included primarily to provide phenotypic context for the transcriptomic and metatranscriptomic analyses.

**Figure 1:**
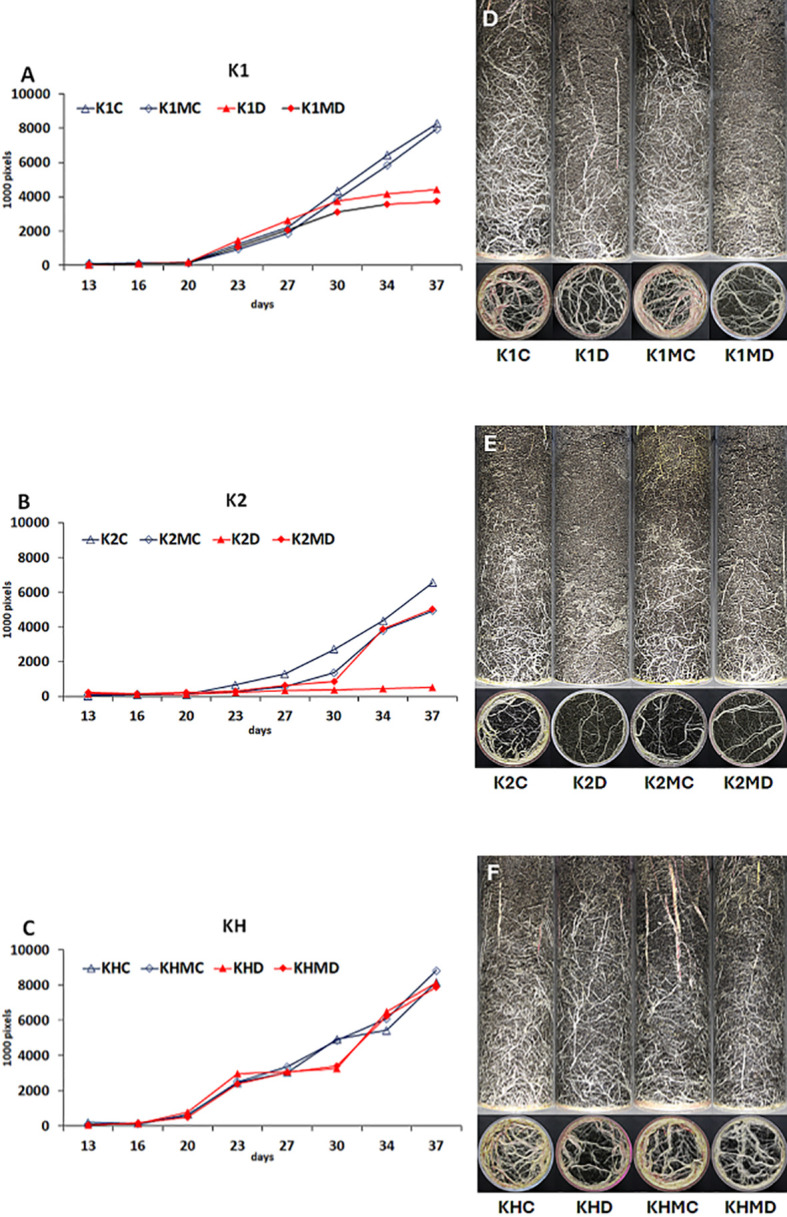
Genotype-dependent variation in early root growth dynamics and root system architecture. **(A–C)** Root growth curves of K1 drought-tolerant genotype, K2 drought-sensitive genotype, and KH: K1 × K2 hybrid, respectively, illustrating distinct temporal elongation patterns during early development. **(D–F)** Representative root system morphology corresponding to K1 drought-tolerant, K2 drought-sensitive, and KH: K1 × K2 hybrid. K1 drought-tolerant exhibits enhanced primary root elongation and a more extensive lateral root network, whereas K2 drought-sensitive shows reduced root length and branching. KH: K1 × K2 hybrid displays intermediate or integrative morphological characteristics. Quantitative growth kinetics and morphological observations reveal clear genotype-dependent differences in early root development that may contribute to differential drought adaptation and resource acquisition efficiency. M, *F. mosseae*-colonized plants; D, drought (30% MC); C, control (60% MC).

Early root development showed pronounced genotype-dependent differences in both elongation dynamics and root system architecture (**Figure 1**). K1 drought-tolerant genotype displayed the most rapid increase in root length over time, accompanied by a well-developed lateral root network (**Figures 1A, D**). In contrast, K2 drought-sensitive genotype exhibited slower elongation kinetics and a less branched root system, indicating reduced intrinsic early root vigor under non-mycorrhizal conditions (**Figures 1B, E**). Importantly, the mycorrhizal treatment resulted in a comparatively stronger stimulatory effect in K2 drought-sensitive plants than in K1 drought-tolerant plants. In K2, mycorrhiza markedly enhanced root elongation and branching relative to the corresponding non-inoculated condition, partially compensating for the otherwise limited early root growth. By comparison, K1 showed only moderate additional stimulation by mycorrhiza, consistent with its already high root development. The KH: K1 × K2 hybrid exhibited intermediate growth dynamics and morphology, as well as an intermediate magnitude of mycorrhizal response, reflecting the combined influence of the parental genotypes (**Figures 1C, F**). Visual assessment of representative root systems supported the quantitative growth patterns, confirming that enhanced elongation was associated with increased lateral root formation and overall root system expansion.

Root colonization by *F. mosseae* varied among maize genotypes and was influenced by soil moisture (**Table 1**). Under drought conditions (30% MC), the drought-tolerant genotype K1 exhibited the highest colonization (62.5%), which was significantly greater than that of the hybrid KH (51.3%) but not statistically different from the drought-sensitive genotype K2 (55.7%). In well-watered control conditions (60% MC), colonization was generally lower, with K1 showing 43.2%, K2 49.5%, and KH 46.3%, with no significant differences among genotypes. These results indicate that drought stress enhanced AMF root colonization in the drought-tolerant K1 genotype, while colonization in K2 and KH remained relatively stable across moisture levels.

**Table 1:** The experimental design.

MC	Genotype	Comparison	Transcriptome	Biological focus
30%	K1	K1MD vs K1D	Root (host)	AMF-mediated modulation of drought stress responses
30%	K2	K2MD vs K2D	Root (host)	Genotype-dependent AMF-induced stress sensitivity
30%	KH	KHMD vs KHD	Root (host)	Holobiont-level integrated root response
60%	K1	K1MC vs K1C	Root (host)	Constitutive mycorrhizal metabolic effects
60%	K2	K2MC vs K2C	Root (host)	Genotype-dependent constitutive response
60%	KH	KHMC vs KHC	Root (host)	Hybrid constitutive root response
30%	All	MD vs D	Meta-TX	Mycorrhiza-associated microbial functional response

The study addresses how *F. mosseae* symbiosis reshapes root and microbial transcriptional programs during drought stress. MC, soil moisture content; M, *F. mosseae*-colonized plants; D, drought (30% MC); C, control (60% MC); K1, drought-tolerant maize parental inbred line; K2, drought-sensitive maize parental inbred line; KH, hybrid maize genotype (KH: K1 × K2).

### 3.2 PCA and DEGs of *F. mosseae*-colonized root transcriptomes

**Figure 2:**
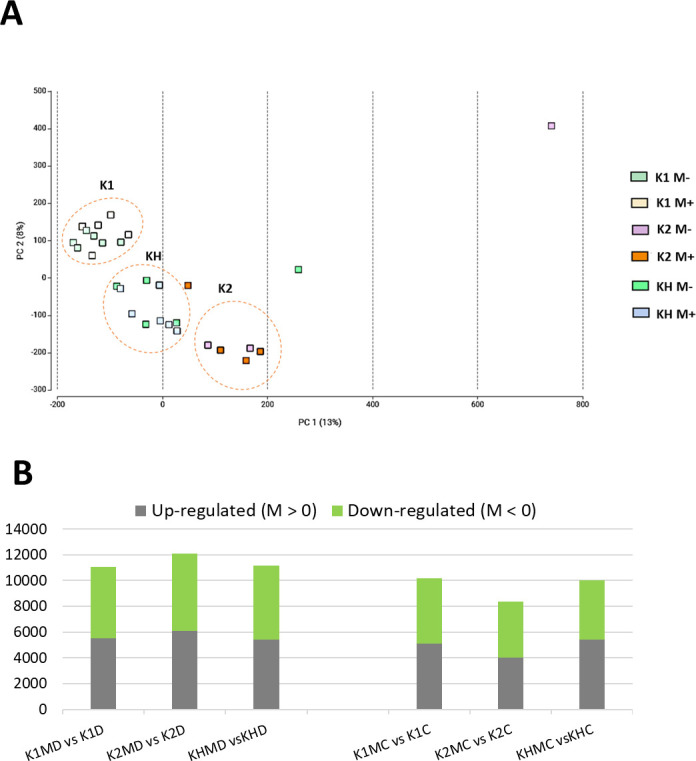
Genotype- and mycorrhiza-dependent variation in global transcriptomic profiles. **(A)** PCA of transcriptome datasets showing clear separation among K1: drought-tolerant, K2: drought-sensitive, and KH: K1 × K2 hybrid genotypes, as well as distinct clustering of mycorrhizal (M+) and non-mycorrhizal (M−) samples. The distribution of samples along PC1 and PC2 indicates strong genotype effects accompanied by a measurable mycorrhiza-induced shift, particularly pronounced in the drought-sensitive background. **(B)** Number of DEGs in response to mycorrhizal colonization across genotypes and conditions. Bars represent up-regulated (M > 0) and down-regulated (M < 0) transcripts in M+ versus corresponding M− comparisons. The magnitude of transcriptional reprogramming varies among genotypes, with the drought-sensitive genotype showing a prominent AMF-associated response, while the hybrid exhibits an intermediate pattern.

Principal component analysis (PCA) of root transcriptomic profiles revealed that genotype was the primary source of variation, with the first principal component (PC1) explaining 13% of the total variance (**Figure 2A**). PC1 separated the K2, drought-sensitive genotype from the K1, drought-tolerant genotype, while the KH hybrid genotype occupied an intermediate position. The second principal component (PC2), explaining 8% of the variance, reflected additional variation associated with mycorrhizal status. Given the relatively low proportion of variance explained by PC1 and PC2, PCA results were interpreted as indicative of global trends rather than strict group separation.

Differential expression analysis (FDR ≤ 0.05, |log_2_;FC| ≥ 1) revealed substantial transcriptional reprogramming in response to mycorrhizal colonization (**Figure 2B**). Under drought conditions, the K2, drought-sensitive genotype exhibited the highest number of differentially expressed genes (DEGs), with 6,082 upregulated and 6,038 downregulated genes (total: 12,120 DEGs). In comparison, K1, drought tolerant genotype showed 5,515 upregulated and 5,564 downregulated genes (total: 11,079 DEGs), while the KH hybrid displayed 5,404 upregulated and 5,752 downregulated genes (total: 11,156 DEGs).

Under control conditions, K1 exhibited 5,120 upregulated and 5,046 downregulated genes (total: 10,166 DEGs), K2 showed 4,002 upregulated and 4,390 downregulated genes (total: 8,392 DEGs), and KH displayed 5,442 upregulated and 4,581 downregulated genes (total: 10,023 DEGs).

These results demonstrate genotype-dependent differences in the extent of transcriptional responses to mycorrhizal colonization, with consistently higher DEG numbers observed under drought compared to control conditions.

The observed variation was further supported by PERMANOVA analysis, which confirmed that host genotype was the dominant determinant of global root transcriptomic structure (pseudo-F = 3.53, R² = 0.207, p = 0.005). In contrast, water regime and mycorrhizal status did not show significant independent effects in the global model (p > 0.05), and no significant interaction effects were detected. These results support the PCA-based observation that genotype represents the primary source of transcriptomic variation, while AMF-induced responses are more genotype-specific and reflected primarily at the differential gene expression level rather than as large-scale shifts in global transcriptomic structure. All DEGs with log2 fold changes, adjusted p-values, and gene annotations for all six pairwise comparisons are available in **Supplementary Material 1**.

### 3.3 Pathway analysis of *F. mosseae*–induced root transcriptomic responses under drought and control conditions

**Figure 3:**
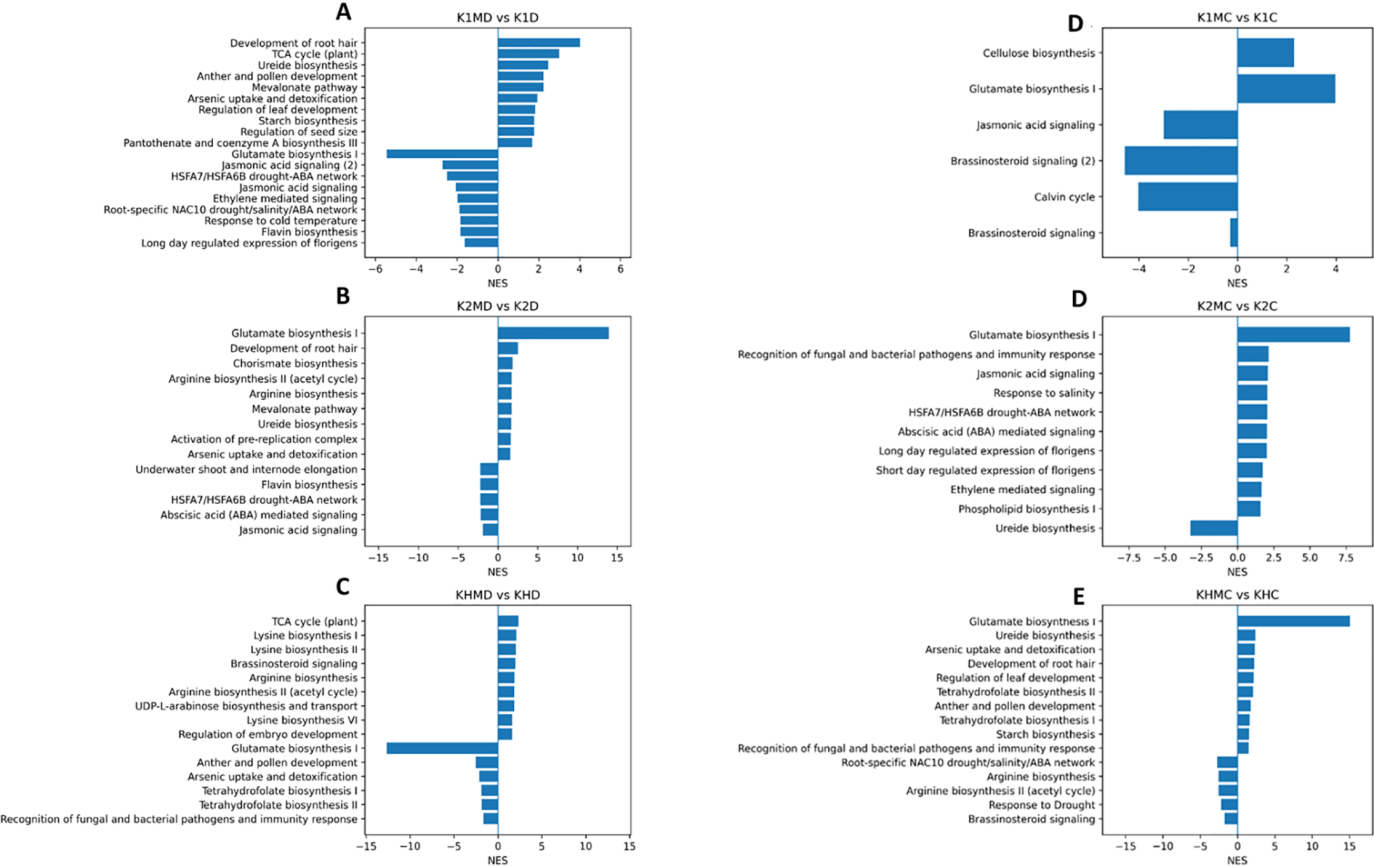
Pathway analysis of root transcriptomes based on plant reactome database. Pathway analysis illustrates genotype-dependent pathway regulation in response to *F. mosseae* colonization under drought stress [**(A, B, C)**, 30% MC] and well-watered control conditions [**(D, E, F)**, 60% MC]. Normalized enrichment scores (NES) are shown for pairwise comparisons between mycorrhizal (M^+^) and non-mycorrhizal (M^-^) roots in the K1: drought-tolerant genotype **(A, D)**, K2: drought-sensitive genotype **(B, E)**, and their KH hybrid genotype **(C, F)**. Panels **(A–C)** represent drought stress conditions, whereas panels **(D–F)** correspond to well-watered control conditions. Positive NES values indicate pathways enriched in mycorrhizal roots, reflecting transcriptional activation or up-ranking of pathway genes in response to mycorrhizal colonization, while negative NES values indicate pathways enriched in non-mycorrhizal roots, corresponding to transcriptional responses that are relatively suppressed or less active in mycorrhizal plants. Under drought stress, mycorrhizal colonization is associated primarily with attenuation of stress- and hormone-related signaling and enrichment of adaptive metabolic pathways, whereas under control conditions it predominantly modulates constitutive metabolic and developmental processes in a genotype-dependent manner, with the hybrid genotype exhibiting the strongest constitutive transcriptional reprogramming.

Pathway enrichment analysis based on the Plant Reactome database revealed genotype-dependent functional responses to *F. mosseae* colonization under both drought (30% MC) and control (60% MC) conditions (**Figure 3**). Enrichment results were considered significant at FDR ≤ 0.05.

Under drought conditions (**Figures 3A–C**), a total of 14–16 significantly enriched pathways were identified per genotype. In the K1, drought-tolerant genotype, the number of negatively enriched pathways exceeded positively enriched ones, with stress-related hormonal pathways (ABA, JA, ethylene signaling) showing negative enrichment (NES range: −5.0 to −1.5), while positively enriched pathways included central metabolic processes such as the TCA cycle, coenzyme A biosynthesis, and starch metabolism (NES range: 1.5 to 4.5).

In the K2, drought-sensitive genotype, 14 significantly enriched pathways were identified, including both stress-related signaling pathways (ABA, JA, heat-shock factor networks) and metabolic pathways. Positively enriched pathways included amino acid metabolism (e.g., glutamate and arginine biosynthesis), while several stress-associated pathways remained negatively enriched (−12.0 to +13.0).

The KH hybrid genotype showed 16 enriched pathways, with both positive and negative enrichment patterns. Positively enriched pathways included energy metabolism, amino acid biosynthesis, and developmental processes, while negatively enriched pathways included defense-related and detoxification pathways (−12.0 to +12.0).

Under control conditions (**Figures 3D–F**), fewer pathways were significantly enriched (6–16 pathways per genotype). In K1, enriched pathways were primarily related to primary metabolism, including glutamate and cellulose biosynthesis, while hormone-related pathways showed negative enrichment (NES range: −4.0 to +5.0). In K2, both stress-related and metabolic pathways were enriched, including ABA, JA, and ethylene signaling pathways (NES range: −7.0 to +8.0). The KH hybrid exhibited the highest number of enriched pathways under control conditions, including metabolic and developmental pathways such as starch biosynthesis and root development (NES range: −10.0 to +15.0).

To summarize, pathway enrichment analysis revealed genotype-dependent differences in the number and direction of enriched pathways in response to mycorrhizal colonization under both environmental conditions.

Comparison between (**Table 2**) water regimes demonstrated that mycorrhizal colonization shifts root functional programs from stress buffering and adaptive metabolic reconfiguration under drought toward growth- and maintenance-oriented metabolic modulation under control conditions. Drought responses were characterized by repression of hormone- and stress-associated transcriptional networks alongside enrichment of central carbon, amino acid, and nitrogen metabolism and root architectural pathways, whereas control conditions primarily involved constitutive metabolic and developmental regulation with limited activation of stress signaling.

**Table 2:** Root mycorrhizal colonization (%) in different genotypes under drought (30% MC) and control (60% MC) conditions.

Maize genotype	Root colonization (%)	
	Drought (30% MC)	Control (60% MC)
K1 drought-tolerant genotype	62.5 ± 7.9 c	43.2 ± 6.4 a
K2 drought-sensitive genotype	55.7 ± 6.2 bc	49.5 ± 2.9 ab
KH: K1 × K2 hybrid	51.3 ± 4.1 ab	46.3 ± 4.9 a

Different regular letters indicate significant differences (p<0.05) among all treatment combinations (genotype, drought) according to Games-Howell post-hoc test of the two-way ANOVA.

These findings indicate that *F. mosseae* induces genotype-specific and environment-dependent root transcriptional strategies, highlighting differential coordination between stress signaling, metabolism, nitrogen utilization, and developmental processes during mycorrhizal symbiosis.

### 3.4 GSEA of root-specific functional differences between drought and control conditions

**Figure 4:**
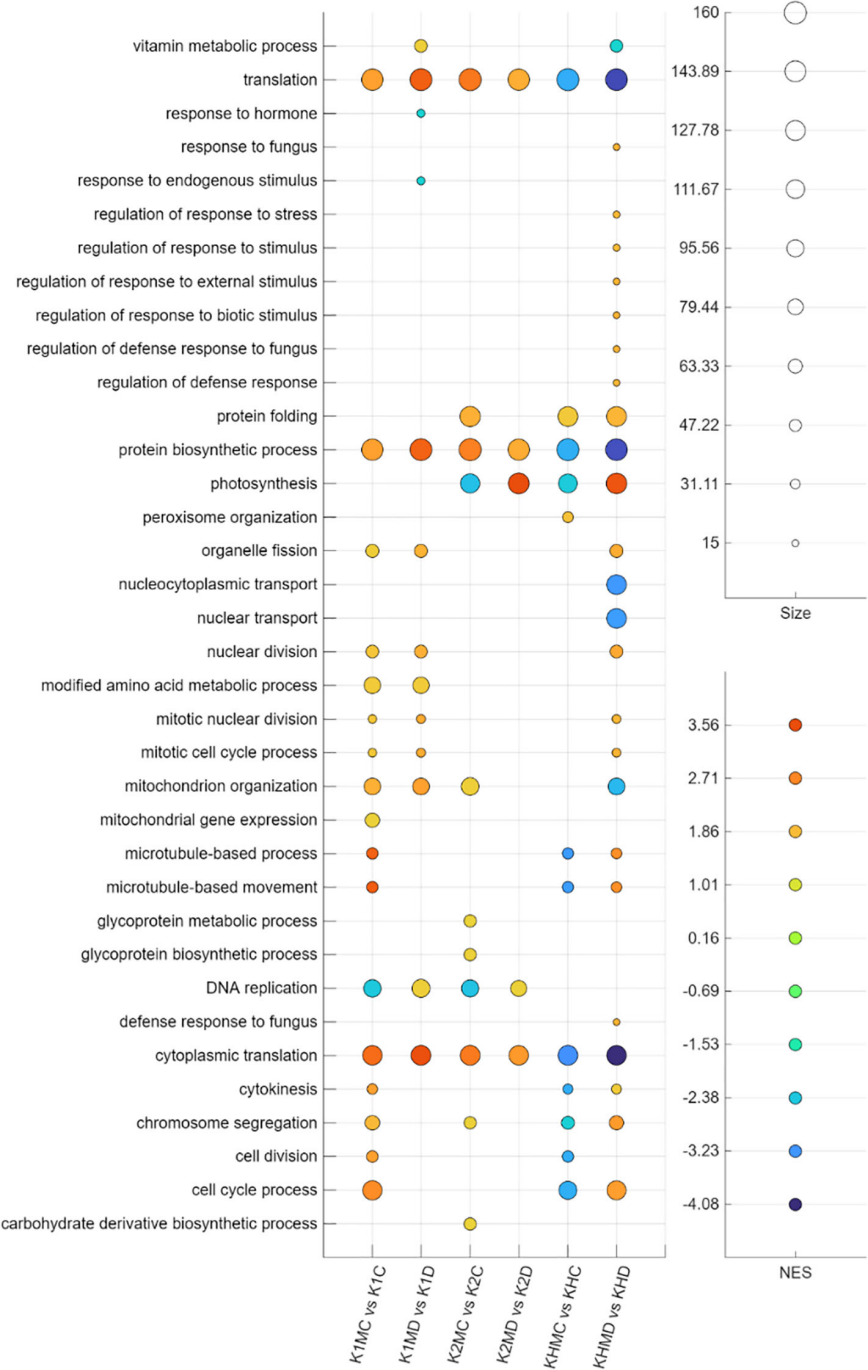
Gene set enrichment patterns across genotypes and treatments visualized by a bubble plot. Bubble size indicates gene set size and color represents NES values. Comparisons include control (30% MC, MC vs. C) and drought (60% MC, MD vs. D) conditions in K1: drought-tolerant, K2: drought-sensitive, and KH: K1 x K2 hybrid maize genotypes. The plot reveals distinct, condition-dependent functional transcriptional states shaped by host genotype and mycorrhizal status.

GSEA revealed pronounced root-specific functional differences between drought stress (30% MC) and well-watered control (60% MC) conditions across genotypes (**Figure 4**). Under drought, *F. mosseae*-associated root responses were characterized by the enrichment of pathways related to protein biosynthesis, mitochondrial organization, amino acid metabolism (notably glutamate and arginine biosynthesis), and stress-related regulatory processes, whereas control conditions predominantly showed modulation of photosynthesis-related functions, cytoskeletal dynamics, and structural cell components. These patterns indicate a shift from growth- and development-oriented programs under control conditions toward metabolic reprogramming and stress-adaptive processes in mycorrhizal roots during drought (**Table 3**).

**Table 3:** Summary of major root-level differences in enriched plant reactome pathways between drought (30% MC, MD vs. D) and well-watered control conditions (60% MC, MC vs. C).

Aspect (root-level)	Drought stress	Control conditions
Dominant response mode	Stress buffering and adaptive reprogramming	Metabolic and developmental modulation
Hormone signaling (ABA, JA, ethylene)	Generally reduced or suppressed	Mildly modulated or genotype-dependent
Stress-related TF networks (NAC10, HSFA7/6B)	Repressed	Weak or absent
Central carbon metabolism (TCA cycle)	Enriched	Slightly enriched or unchanged
Amino acid metabolism (glutamate, arginine)	Strongly enriched	Enriched
Nitrogen-related pathways (ureide biosynthesis)	Enriched, stress-associated	Genotype-dependent, weaker effects
Root architecture (root hair development)	Enriched	Enriched
Growth-related pathways (cellulose, starch biosynthesis)	Minor or secondary	Prominently enriched
Overall functional interpretation	Adaptive, stress-mitigating root strategy	Growth- and maintenance-oriented root program

The table highlights contrasting functional response modes associated with *F. mosseae* colonization under drought and control conditions, including shifts in hormone signaling, stress-related transcriptional networks, central carbon and amino acid metabolism, nitrogen-associated pathways, and root developmental processes.

**Figure 5:**
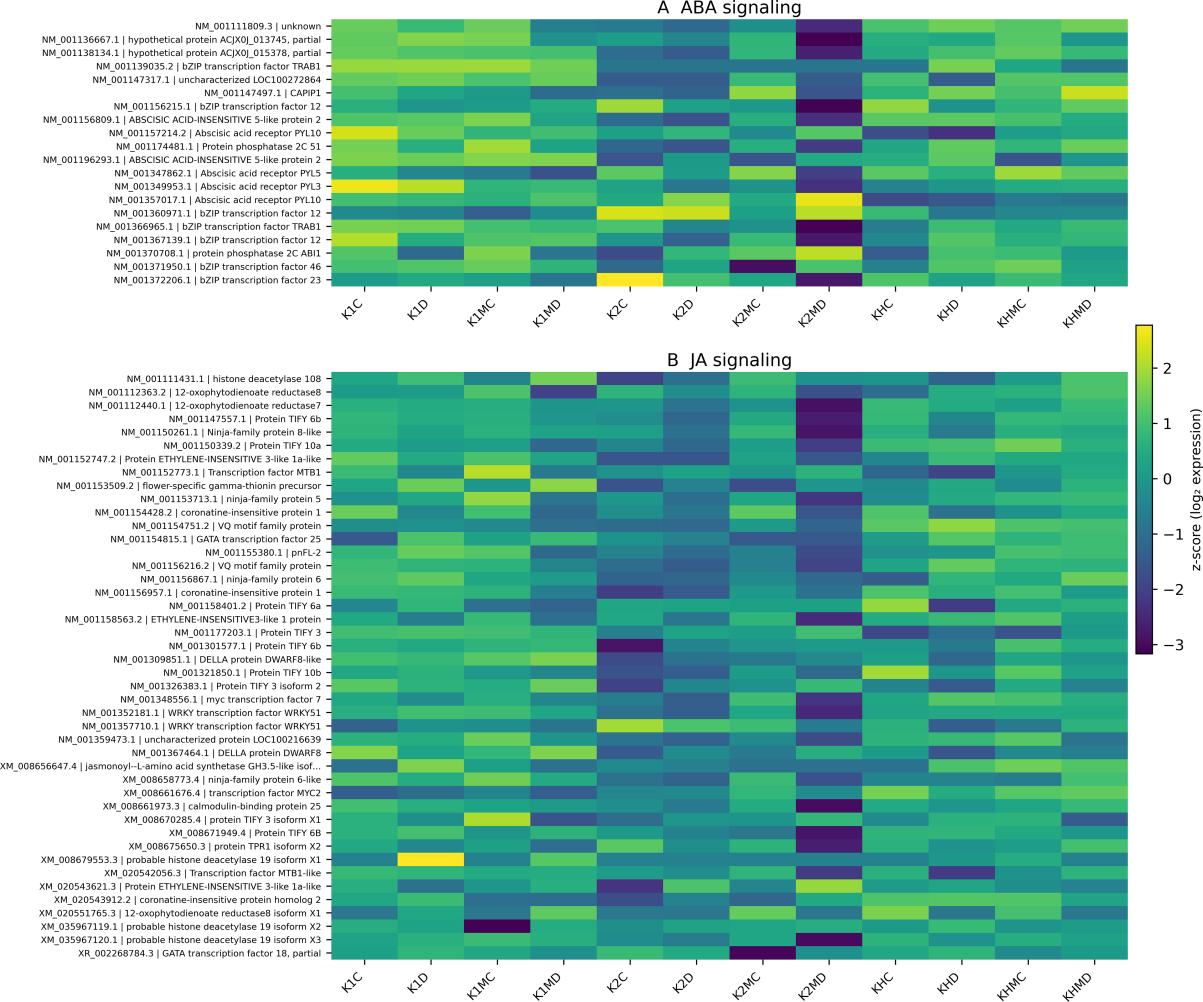
Normalized expression patterns of ABA- and JA-signaling genes across maize genotypes and treatments. Heatmaps show relative transcript abundance of selected genes involved in **(A)** ABA signaling and **(B)** JA signaling in roots of the K1: drought-tolerant, K2: drought-sensitive, and hybrid KH: K1 × K2 genotypes under control **(C)**, drought **(D)**, and mycorrhizal (MC, MD) conditions. Gene expression levels are presented as z-score–scaled log_2_;-transformed RPM (reads per million mapped reads) values, where warmer colors indicate higher relative expression and cooler colors indicate lower expression within each gene. Patterns highlight genotype-dependent regulation and non-additive, heterosis-associated expression in the hybrid, particularly in key signaling nodes of the ABA and JA regulatory networks.

The GSEA bubble plot presents the molecular function GO categories across the samples (**Figure 5**). This analysis reveals a condition- and genotype-dependent functional reorganization in maize roots. Under drought conditions (MD vs. D), multiple genotypes exhibit strong enrichment of translation and protein biosynthesis, cell cycle–related processes, and mitochondrial organization, indicating elevated metabolic activity and cellular reprogramming associated with stress adaptation. Drought stress also promotes enrichment of amino acid metabolic processes, cytoskeletal and microtubule-based processes, and stress- and stimulus-response categories, reflecting coordinated structural and metabolic adjustment at the root level. In contrast, control conditions (MC vs. C) are characterized by relatively stronger enrichment of photosynthesis-related pathways, carbohydrate derivative biosynthetic processes, and selected growth-associated functions, consistent with a maintenance-oriented transcriptional state under optimal water supply. Notably, the magnitude and direction of enrichment differ across genotypes. The K1 drought-tolerant genotype displays a more coordinated enrichment of growth and energy-related pathways under drought, whereas the K2 drought-sensitive genotype shows more heterogeneous shifts, including pronounced changes in photosynthesis- and stress-associated categories. The KH: K1 x K2 hybrid genotype exhibits an intermediate yet distinct enrichment profile, combining features of metabolic activation, protein folding, and defense-related processes. The distribution of NES scores across functional categories supports the presence of discrete, condition-specific transcriptional states rather than uniform quantitative changes, with host genotype and mycorrhizal status jointly shaping root functional responses. Cellular component and biological process enriched categories are presented in **Supplementary Figures 1**, **2**.

### 3.5 Gene expression analysis of hormone signaling, root hair development, arbuscular mycorrhizae-related genes, and lateral root genes

*ABA and JA hormone signaling genes*

**Figure 6:**
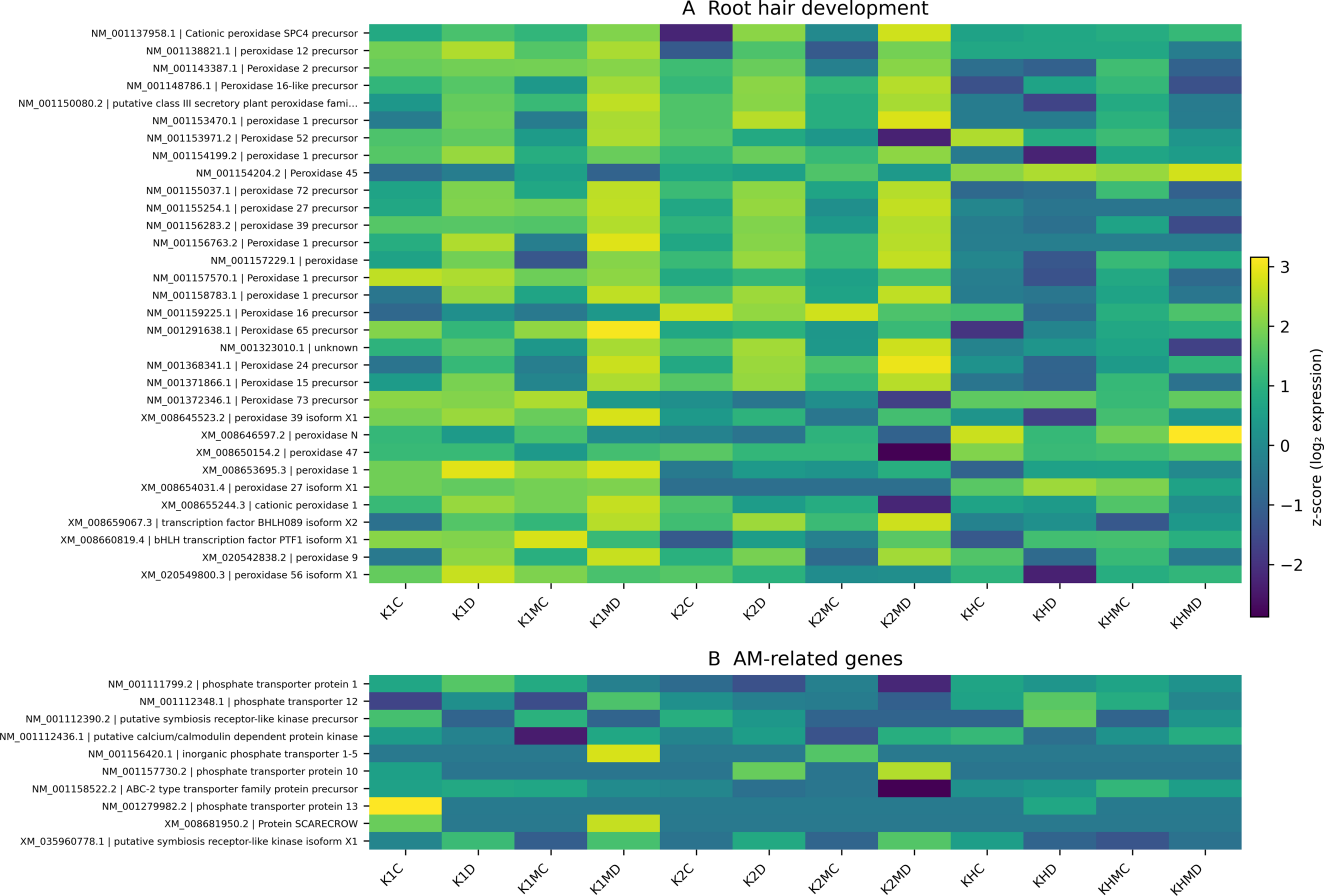
Normalized expression patterns of genes involved in root hair development and Arbuscular mycorrhizal–related functions. Heatmaps display relative transcript abundance of selected genes associated with **(A)** root hair formation—including class III peroxidases and regulatory transcription factors—and **(B)** AM-related signaling and phosphate transport in roots of the K1 drought-tolerant, K2 drought-sensitive, and KH: K1 × K2 hybrid genotypes under control **(C)**, drought **(D)**, and mycorrhizal (MC, MD) conditions. Gene expression values are shown as z-score–scaled log_2_;-transformed RPM, where warmer colors indicate higher and cooler colors lower relative expression within each gene. Patterns reveal genotype-dependent regulation and heterosis-associated enhancement of root developmental remodeling and symbiotic nutrient acquisition in the hybrid.

Gene expression patterns of ABA and JA signaling pathways, root hair development, and AM-related genes are shown in **Figures 5**, **6** as log_2_;-transformed RPM values. Only genes showing consistent expression patterns across biological replicates are described.

ABA signaling genes (**Figure 5A**) exhibited genotype-dependent expression patterns under drought and mycorrhizal conditions. The ABA receptor PYL10 showed consistently high expression across treatments, with slightly reduced levels in mycorrhizal K1 compared to non-mycorrhizal samples, while remaining elevated in K2 and showing intermediate values in the KH hybrid. Among PP2C phosphatases, PP2C51 displayed strong expression under drought conditions across all genotypes, whereas ABI1 showed comparatively moderate levels. The transcription factor bZIP23 exhibited consistently high expression under drought, particularly in K2 and KH, while additional bZIP12 and TRAB1 isoforms showed variable expression patterns across genotypes. Overall, ABA-related genes showed higher expression levels in K2 and more moderate or intermediate expression in K1 and KH.

JA signaling genes (**Figure 5B**) also showed clear genotype-dependent differences. The receptor component COI1 and its homologs were highly expressed under drought conditions, with higher levels in K2, moderate levels in K1, and intermediate values in KH. Several JAZ/TIFY family members (including TIFY10a, TIFY3, and TIFY10b) displayed strong expression across treatments, consistent with their role as transcriptional repressors. Downstream regulators such as EIN3-like, MYC2, and NINJA exhibited sustained expression across genotypes, with KH generally showing intermediate expression relative to parental lines. These results indicate that JA signaling components are broadly activated under drought, with genotype-dependent variation in expression magnitude.

#### 3.5.1 Root hair development and AM-related genes

Individual gene expression profiles of root hair development (**Figure 6A**) genes revealed strong, drought-responsive induction of multiple class III peroxidases (e.g., *peroxidase 1, 16, 24, 27, 52, and 72*) together with regulatory transcription factors such as bHLH family members. In the K1: drought-tolerant genotype, mycorrhizal colonization generally moderated peroxidase expression, indicating controlled cell wall remodeling and stabilized root surface differentiation. In contrast, the K2: drought-sensitive genotype maintained elevated expression of several peroxidase isoforms under stress, consistent with continued structural adaptation. The KH: K1 x K2 hybrid frequently showed intermediate-to-enhanced expression across key peroxidases and *bHLH* regulators, suggesting heterosis-associated optimization of root hair formation that may improve soil exploration and symbiotic competence.

Within AM-related genes (**Figure 6B**), phosphate transporters and signaling components displayed clear genotype-dependent behavior. Phosphate transporter 1 (*PT1*) and *phosphate transporter 13* showed strong expression, while *inorganic phosphate transporter 1–5* and *phosphate transporter 10* were selectively induced under mycorrhizal drought conditions. Symbiosis-associated regulators, including a *symbiosis receptor-like kinase*, *calcium/calmodulin-dependent protein kinase*, and *SCARECROW*, further supported active AM signaling. Notably, the KH: K1 x K2 hybrid often matched or exceeded the higher parental expression for transport- and signaling-related genes, indicating heterotic enhancement of nutrient uptake and symbiotic responsiveness. The gene-level patterns link peroxidase-driven root hair remodeling with AM-mediated phosphate acquisition, and demonstrate that the KH: K1 x K2 hybrid integrates parental traits through non-additive regulation that supports improved drought-adaptive root function.

#### 3.5.2 Lateral root development genes

**Figure 7:**
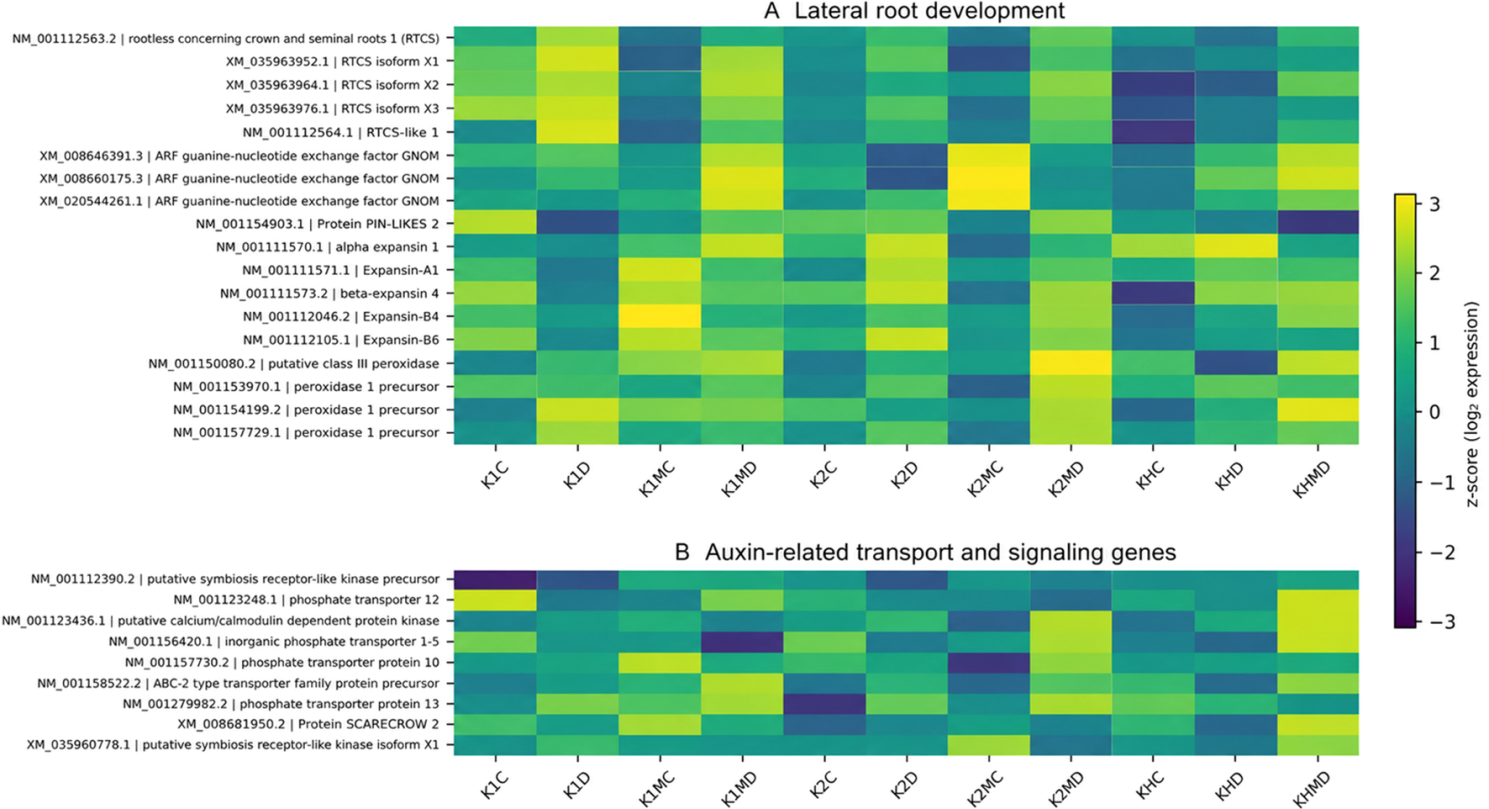
Normalized expression patterns of genes involved in lateral root development and auxin-related transport and signaling genes. Heatmap showing z-score normalized log2 transcript abundance of genes associated with lateral root development and auxin- signaling across maize genotypes and treatments. **(A)** Canonical lateral root developmental regulators and associated structural remodeling genes, including RTCS (rootless concerning crown and seminal roots 1), RTCS-like, GNOM auxin trafficking regulators, PIN-LIKES 2 auxin transporter, expansins, and class III peroxidases. **(B)** Auxin-signaling involves root developmental signaling and symbiosis/nutrient-responsive genes, including phosphate transporters, SCARECROW2, calcium/calmodulin-dependent kinase, and symbiosis receptor-like kinases. Yellow indicates relatively higher expression, whereas blue/purple indicates lower expression. The data reveal genotype-specific transcriptional alteration of root developmental pathways under drought stress and AM colonization, with pronounced induction of lateral root-associated genes in drought-treated K1 plants and strong remodeling responses in mycorrhizal K2 and hybrid samples.

Transcriptomic analysis of genes associated with lateral root development revealed clear genotype- and treatment-dependent expression patterns (**Figure 7**). Among the key maize root developmental regulators, RTCS (rootless concerning crown and seminal roots 1) (NM_001112563.2) showed marked induction in drought-treated K1 plants, with the highest expression observed in K1D and KHMD samples (**Figure 7A**). A similar expression pattern was detected for RTCS-like 1 (NM_001112564.1), which exhibited particularly strong upregulation in the KHMD group. Genes involved in auxin-mediated regulation of root architecture also displayed differential expression across treatments (**Figure 7B**). The GNOM ARF guanine-nucleotide exchange factor homologs (XM_008646391.3, XM_008660175.3, XM_020544261.1) showed dynamic transcriptional variation among the experimental groups, while the PIN-LIKES 2 auxin transporter (NM_001154903.1) also exhibited treatment-dependent expression changes. Transcripts associated with root emergence and cell wall remodeling were strongly represented among the responsive genes. Several expansin family members, including alpha-expansin 1 (NM_001111570.1), Expansin-A1 (NM_001111571.1), beta-expansin 4 (NM_001111573.2), Expansin-B4 (NM_001112046.2), and Expansin-B6 (NM_001112105.1), showed elevated expression, particularly in drought-treated and mycorrhizal K1 and K2 samples. Multiple class III peroxidase transcripts also displayed increased expression in several treatment groups. The transcriptional response differed among genotypes. The K1 genotype exhibited the strongest induction of lateral root-associated developmental genes, particularly under drought conditions, whereas the hybrid genotype showed selective activation, with pronounced induction of RTCS-related transcripts but less consistent expression changes among expansin-associated genes. This targeted analysis identified several genes directly or indirectly associated with lateral root development, including canonical maize root developmental regulators, auxin transport-related genes, and cell wall remodeling-associated transcripts.

### 3.6 Global analysis of root-associated metatranscriptome

**Figure 8:**
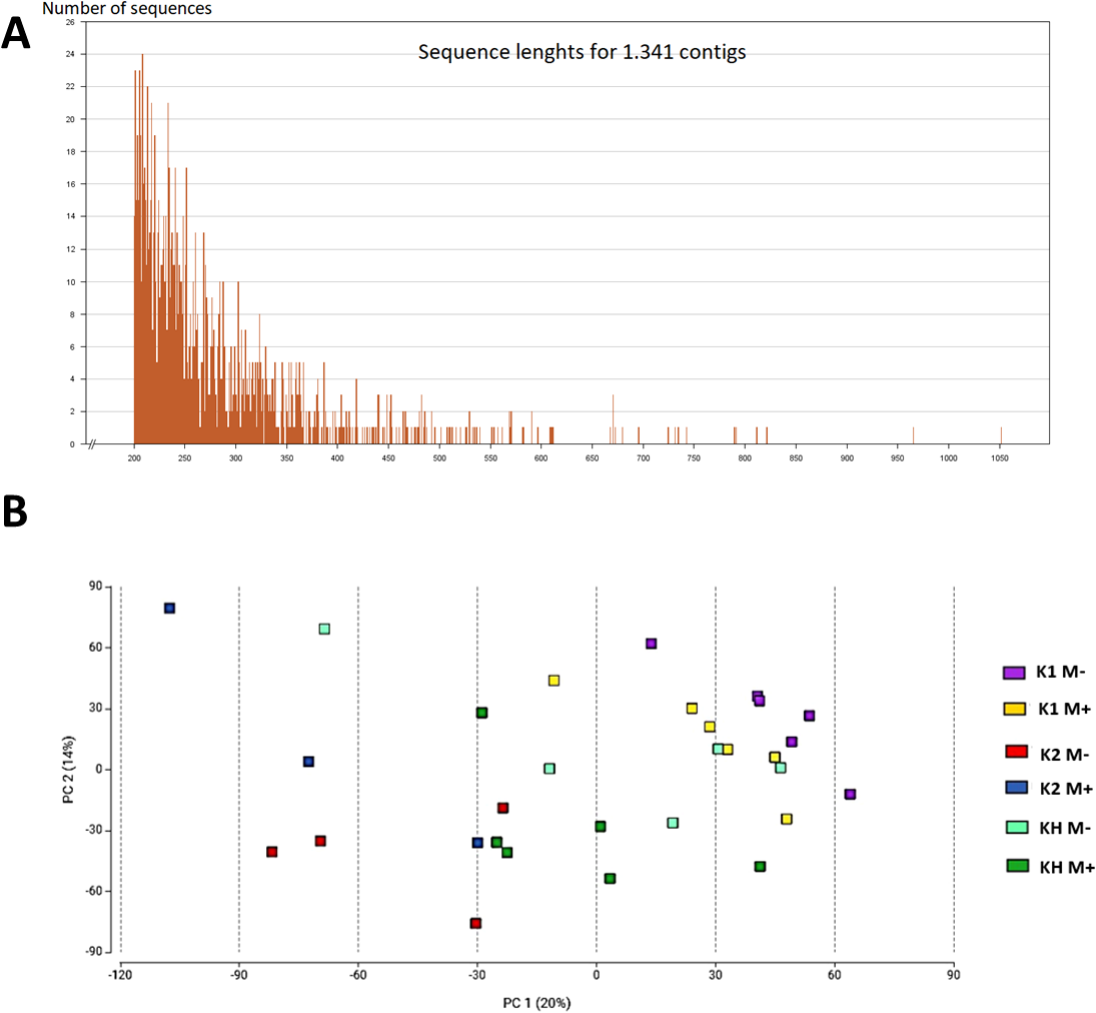
Structural and functional organization of the root-associated metatranscriptome under AMF-mediated drought stress. *De novo* assembly of host-filtered reads **(A)** produced 1,341 contigs predominantly 200–300 bp in length, with longer contigs (>500 bp) indicating successful reconstruction of abundant microbial transcripts. PCA of normalized metatranscriptomic expression profiles **(B)** revealed genotype- and symbiosis-driven structuring of microbial functional activity (PC1, 20%) and treatment-dependent variation (PC2, 14%). The intermediate clustering of the KH: K1 x K2 hybrid supports a partially integrated holobiont response shaped by host genetic background and mycorrhizal interaction.

*De novo* metatranscriptomic assembly of host-depleted reads resulted in 1,341 contigs (**Figure 8A**), with a length distribution skewed toward shorter sequences (200–300 bp), consistent with the high diversity and variable expression levels of microbial transcripts. The presence of contigs exceeding 500 bp suggests successful reconstruction of longer, high-abundance transcripts suitable for downstream functional annotation.

The PCA analysis of transcript abundancies of the root-associated metatranscriptome revealed that PC1 (20% of the variance) primarily separates samples according to the combined effect of host genotype and mycorrhizal status. In contrast, PC2 (14%) captures treatment-dependent variation, reflecting differences in microbial activity associated with mycorrhizal colonization and, to a lesser extent, drought-related stress responses (**Figure 8B**). A clear genotype-specific microbial response was observed, as samples from K1, K2, and KH genotypes formed distinct clusters. This indicates that the active root-associated microbial transcriptome is strongly shaped by host genetic background, supporting the concept that AMF-associated microbial responses are not uniform but are structured by host genotype. Across multiple genotypes, mycorrhizal (M+) and non-mycorrhizal (M−) samples showed partial separation, particularly along PC1, suggesting that AMF colonization reprograms not only host transcriptional responses but also the functional activity of the root-associated microbiome. The hybrid genotype (KH) displayed an intermediate yet distinct positioning between the parental genotypes, consistent with an integrated holobiont-level functional response pattern rather than a simple additive or intermediate response. Finally, the relatively high dispersion of samples in the metatranscriptomic PCA compared with host RNA-seq analyses reflects the inherently dynamic and heterogeneous nature of microbial functional activity, a characteristic feature of metatranscriptomic datasets.

### 3.7 GSEA of root-associated metatranscriptome

GSEA based GO annotations revealed structured differences among the different genotype metatranscriptomic profiles, defining three discrete functional transcriptional states (**Supplementary Material 1**).

The KHMD vs KHD sample was characterized by a predominance of negative enrichment, with multiple GO terms showing large-magnitude negative NES and false discovery rate–corrected significance (FDR < 0.05). Enrichment signals were broadly distributed across biological process, cellular component, and molecular function ontologies, indicating coordinated transcriptional repression. In contrast, the K1MD vs K1D sample exhibited positive enrichment of GO terms, with elevated NES values and statistically supported enrichment in multiple functional categories. Enrichment patterns in K1 were pathway-selective rather than global, consistent with regulated transcriptional activation. The K2MD vs K2D sample displayed a low-amplitude, bidirectional enrichment profile, with NES values centered near zero and no GO terms passing the FDR < 0.05 threshold. Despite the lack of statistical significance, the directional consistency of NES values indicates a biologically coherent transcriptional state.

Within the biological process domain, KHMD vs KHD showed significant negative enrichment of gene sets associated with metabolic process, small-molecule metabolism, generation of precursor metabolites and energy, and nucleobase-containing compound metabolic process, reflecting global downregulation of energy- and resource-intensive pathways. K1MD vs K1D, in contrast, demonstrated positive enrichment of nucleobase-containing compound metabolic process, carbohydrate derivative metabolic process, and transmembrane transport, indicating activation of metabolic and transport-related processes linked to transcriptional and biosynthetic activity. K2MD vs K2D exhibited intermediate behavior, with near-neutral NES values for core metabolic processes, consistent with maintenance-level metabolic activity in the absence of pathway-specific amplification.

Cellular component enrichment patterns further differentiated the samples. KHMD vs KHD showed strong negative enrichment of mitochondrion, plastid, cytoplasm, and intracellular organelle gene sets, indicating reduced transcriptional investment in cellular architecture and energy-associated compartments. K1MD vs K1D displayed positive enrichment of membrane-bounded organelles, mitochondrion, and plastid, consistent with enhanced organellar activity and metabolic engagement. K2MD vs K2D retained moderate positive NES values for cytoplasm, ribosome, and related cellular structures, suggesting preservation of basal cellular organization without extensive structural remodeling.

**Figure 9:**
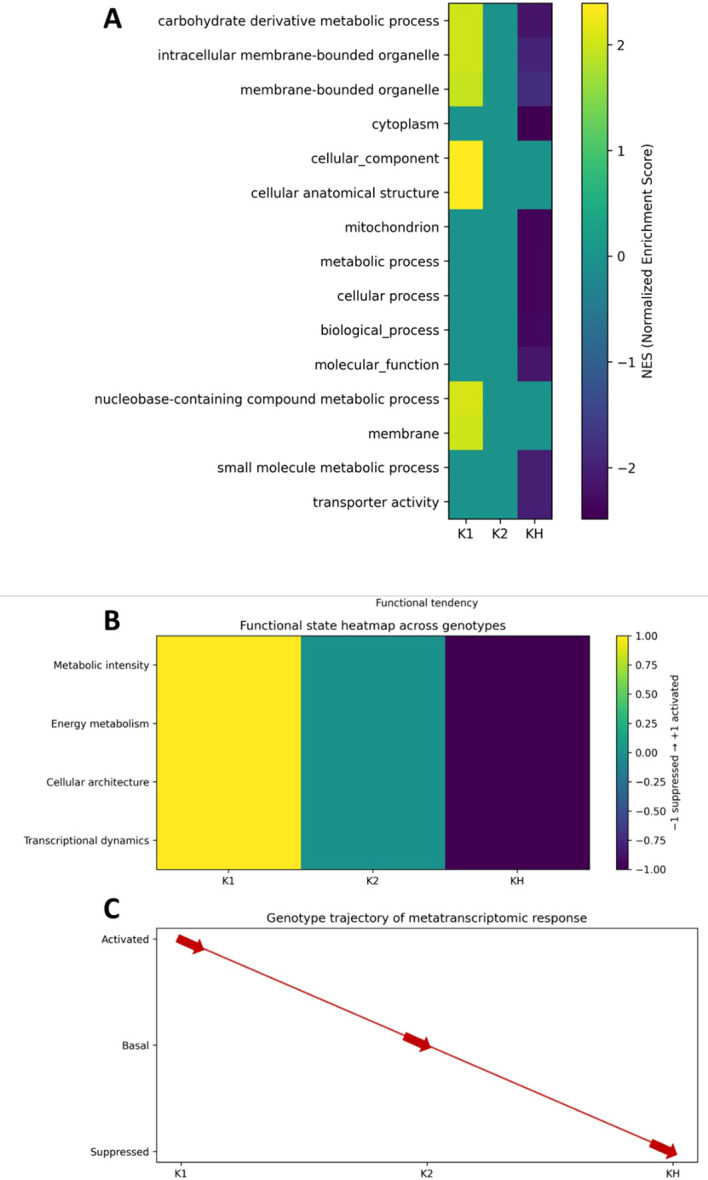
Genotype-dependent holobiont functional organization of the root-associated metatranscriptome under AMF-mediated drought. GSEA–based heatmap **(A)** showing normalized enrichment scores (NES) of major functional categories across maize genotypes. The pattern indicates selective metabolic activation in the K1: drought-tolerant, intermediate or compensatory activity in the K2: drought-sensitive, and broad functional suppression in the KH: K1 x K2 hybrid. Conceptual functional-state heatmap **(B)** summarizing genotype-dependent tendencies in metabolic intensity, energy metabolism, cellular architecture, and transcriptional dynamics, highlighting activated states in K1, basal states in K2, and suppressed states in KH. Genotype trajectory model **(C)** illustrating the progressive shift from activated microbial functional organization (K1) through basal coordination (K2) toward suppressed holobiont activity in the hybrid (KH), consistent with genotype-specific AMF-mediated drought response strategies.

In the molecular function category, KHMD vs KHD exhibited negative enrichment of catalytic activity, transporter activity, and oxidoreductase activity, indicating suppression of enzymatic turnover and transmembrane exchange. K1MD vs K1D showed positive enrichment of RNA binding, nucleic acid binding, and catalytic activity, consistent with increased transcriptional dynamics and enzymatic function. K2MD vs K2D showed near-neutral enrichment of binding-related molecular functions, with slight negative tendencies in redox-associated activities, indicating functional stability without enhanced stress or detoxification responses. **Figure 9** represents the main GSEA categories (**Figure 9A**), genotype-dependent tendencies in metabolic intensity, energy metabolism, cellular architecture and transcriptional dynamics (**Figure 9B**) and genotype trajectory model (**Figure 9C**).

## 4 Discussion

Drought stress is a major constraint on maize productivity, with phosphorus (P) availability, root function, and plant–microbe interactions jointly determining drought tolerance. Because phosphate diffusion in soil is severely limited under drought, efficient regulation of phosphate uptake pathways is critical for plant adaptation (Sawers et al., 2017). In maize, members of the PHT1 phosphate transporter family play central roles in both direct and mycorrhiza-mediated phosphate acquisition, and their expression is strongly influenced by phosphate availability, mycorrhizal colonization, and environmental conditions. Previous studies have shown that AM-inducible transporters such as *Pht1;6* are essential for symbiotic phosphate uptake and AM colonization, with loss-of-function mutants displaying reduced biomass and P acquisition under low-P conditions (Willmann et al., 2013; Sawers et al., 2017). Similar mycorrhiza-responsive phosphate transport mechanisms have been reported in other species, including tomato, where phosphate transporters induced by AM colonization act synergistically under drought stress (Szentpéteri et al., 2024).

In our study, drought induced a consistent repression of direct phosphate uptake transporters across genotypes that is presented in pathway enrichment analysis (**Figures 3A–C**) and in gene expression analysis (**Figure 6B**), suggesting a functional shift from direct root uptake toward AM-mediated phosphate acquisition under conditions of limited soil phosphate mobility. Such transcriptional repression of direct uptake pathways has been reported as a strategy to optimize carbon allocation and avoid redundant phosphate acquisition mechanisms under stress (Sawers et al., 2017), this interpretation is based on transcriptomic evidence and does not directly demonstrate functional flux changes. Some studies report concurrent activation of both direct and mycorrhizal pathways under severe nutrient limitation, indicating that this balance may depend on genotype and environmental context.

Notably, phosphate transporter 12 (*PT12*) displayed a strongly genotype-dependent response under mycorrhizal drought (**Figure 6B**). *PT12* expression was induced exclusively in the K1: drought-tolerant genotype, whereas it declined in the K2: drought-sensitive genotype and in the KH hybrid, suggesting that *PT12* may contribute to drought-adaptive phosphate management rather than acting as a general uptake transporter.

One alternative explanation is that the observed repression of direct phosphate transporters reflects a general drought-induced resource reallocation strategy rather than a strictly AMF-specific effect. However, the consistent differences between mycorrhizal (M^+^) and non-mycorrhizal (M^-^) plants (**Figures 3A–C**) within the same genotype and treatment support the interpretation that AMF colonization actively modulates phosphate acquisition strategies rather than merely reflecting stress responses (see ABA/JA expression in **Figures 5A, B**).

One possible explanation is that *PT12* may contribute to the intracellular or tissue-specific redistribution of mycorrhiza-derived phosphate, facilitating efficient transfer from cortical cells to the vascular system under drought. Up-regulation of *PT12* in K1 may therefore support sustained phosphate delivery to the shoot and improved physiological performance, whereas repression of *PT12* in K2 and KH may limit phosphate partitioning despite AM colonization. The KH hybrid response further indicates that heterosis does not uniformly enhance all stress-adaptive regulatory mechanisms; while KH exhibits non-additive up-regulation of several transport, metabolic, and signaling pathways, fine-scale phosphate allocation appears to remain genotype-restricted.

Beyond phosphate transport, our transcriptomic analyses demonstrate that arbuscular mycorrhizal symbiosis by *F. mosseae* is associated with genotype-dependent and context-specific modulation of maize root drought responses. In the K1 drought-tolerant genotype, AM colonization was associated with attenuation of canonical stress- and hormone-related signaling pathways, including ABA-, jasmonic acid–, and ethylene-mediated networks, alongside reduced activity of drought-inducible transcription factors such as *NAC10* and *HSFA7/HSFA6B* (**Figures 3A**, **5A, B**). Given the well-established role of jasmonate signaling in stress-adaptive root architectural remodeling, including lateral root development under abiotic stress conditions, this attenuation may reflect reduced stress burden rather than impaired adaptive capacity (Muzaffar et al., 2024). Concurrent enrichment of central metabolic pathways (**Figure 3A**), including the TCA cycle, amino acid biosynthesis, and root developmental processes, is consistent with a stress-buffering mode (**Figure 4**) of AMF-mediated drought adaptation, consistent with earlier physiological and transcriptomic studies (López-Ráez and Pozo, 2013; Ruiz‐Lozano et al., 2016; Begum et al., 2019b; Zhang et al., 2025). While this attenuation of stress signaling agrees with several studies (Begum et al., 2019a; b), contrasting findings have also been reported, where AMF colonization maintained or even enhanced stress-related signaling pathways (Quiroga et al., 2017), suggesting that these responses are highly context- and genotype-dependent.

Importantly, negative GSEA enrichment does not imply complete transcriptional suppression of all components within a signaling pathway, but rather a relative downward shift in the coordinated expression behavior of the full pathway gene set. Accordingly, selected key regulatory genes, such as PYL10, PP2C51, bZIP23, COI1, and TIFY family members, may remain transcriptionally active despite overall attenuation of pathway-wide stress signaling. This pattern likely reflects maintenance of signaling competence within a more tightly regulated and energy-efficient stress adaptation strategy in the K1 drought-tolerant plants.

In contrast, the K2 drought-sensitive genotype retained strong signatures of stress-associated signaling under mycorrhizal drought (**Figures 3B**, **5A, B**), with incomplete suppression of ABA- and JA-related pathways despite enhanced activation of metabolic and nitrogen-related processes (**Figure 4**; **Table 4**). This pattern suggests that AMF- associated responses in sensitive genotypes are dominated by compensatory metabolic activation rather than effective dampening of stress perception, in agreement with previous reports of genotype-dependent limits to mycorrhizal drought mitigation in maize and other cereals (Quiroga et al., 2017; Begum et al., 2019b; Hu et al., 2020; Chang and Lin, 2025).

**Table 4:** Summary of major functional categories identified by GSEA in maize roots under drought and control conditions.

Functional category	Drought stress	Control conditions	Representative GO terms
Protein biosynthesis, translation	Strong enrichment	Weak or genotype-dependent	cytoplasmic translation, protein biosynthetic process
Cell cycle and division	Enriched (K1, K2, KH)	Often suppressed or unchanged	mitotic cell cycle, nuclear division, cytokinesis
Mitochondrial organization, energy metabolism	Enriched	Minor contribution	mitochondrion organization, TCA-related processes
Amino acid metabolism	Strong enrichment	Moderate enrichment	glutamate biosynthesis I, arginine biosynthesis
Stress and stimulus response	Enriched	Weak or absent	response to hormone, response to endogenous stimulus
Defense-related processes	Pronounced in hybrid	Limited	response to fungus, defense response
Photosynthesis-related processes	Suppressed	Strongly enriched	photosynthesis, thylakoid organization
Cytoskeleton and microtubule dynamics	Enriched	suppressed	microtubule-based movement, cytoskeletal motor activity
Cell wall and extracellular structures	Enriched	Genotype-dependent	cell wall, external encapsulating structure
Molecular functions (binding activities)	Antioxidant and stress-related binding	Structural and nucleotide binding	antioxidant activity, nucleotide binding

The table compares dominant biological processes and molecular functions enriched in mycorrhizal roots under drought stress (30% MC, MD vs. D) and well-watered control conditions (60% MC, MC vs. C), highlighting condition-specific shifts in protein biosynthesis, cell cycle regulation, energy metabolism, stress and defense responses, cytoskeletal dynamics, and photosynthesis-related pathways based on representative Gene Ontology (GO) terms.

These observations further highlight that AMF-mediated drought responses are not uniform across genotypes, but instead reflect differences in the capacity of host regulatory networks to integrate symbiotic and stress signals.

The KH hybrid exhibited a distinct and integrated transcriptional response (**Figures 3C**, **6A, B**) combining metabolic adjustment, protein quality control, root remodeling, and defense-related pathways. Enrichment of protein folding, vacuolar and lysosomal components, and pathways (**Figure 4**) related to fungal recognition and defense is consistent with a potential holobiont-level coordination of drought responses, in which host and symbiont processes may be tightly linked (Ait-El-Mokhtar et al., 2023; Chávez‐González et al., 2024). However, the enrichment of defense-related pathways may also reflect a generalized stress or immune activation response, rather than an optimized symbiotic state, indicating that heterosis enhances responsiveness but not necessarily efficiency of AMF-mediated adaptation. While this integrated response reflects heterosis-associated enhancement of multi-pathway coordination, it does not fully recapitulate the optimized phosphate partitioning observed in the K1: drought-tolerant genotype.

Under well-watered conditions, *F. mosseae* effects were largely confined to metabolic and developmental pathways (**Figures 3D–F**), including photosynthesis-related functions, cell wall biosynthesis, and root growth processes, with minimal activation of stress-associated signaling (**Figure 4**). This contrast indicates that the extensive transcriptional reprogramming observed under drought is stress-dependent rather than a constitutive feature of mycorrhizal colonization (Virág et al., 2025).

In addition to the broad enrichment of root developmental pathways, targeted analysis of lateral root-associated transcripts (**Figure 7**) provided further evidence for genotype-specific root architectural reprogramming under drought and AM colonization. Canonical maize developmental regulators, including RTCS and RTCS-like, were strongly induced, particularly in K1 drought-treated plants and in the mycorrhizal drought-treated hybrid, indicating activation of root developmental programs associated with architectural adjustment. Consistently, genes involved in auxin transport and polarity establishment, including GNOM homologs and PIN-LIKES 2, also showed treatment-dependent modulation, suggesting altered auxin-mediated root developmental regulation. In parallel, upregulation of several expansin family members and class III peroxidases indicates active cell wall remodeling and ROS-associated structural adaptation, processes required for root emergence and developmental plasticity. The strongest induction of lateral root-associated regulators in the K1 drought-tolerant genotype suggests a more effective AMF-supported root architectural adaptation, whereas K2 drought-sensitive genotype showed a response dominated by structural remodeling rather than canonical developmental regulation. These findings are consistent with previous reports that AM fungi influence host root architecture through coordinated interactions between auxin signaling, phosphate sensing, and developmental regulatory networks. Similar AMF-mediated modulation of root architectural traits under drought has been reported in maize, where mycorrhizal colonization promoted root branching and improved stress adaptation through coordinated hormonal and developmental regulation (Begum et al., 2019a).

Metatranscriptomic GSEA further revealed that K1, KH, and K2 represent distinct functional transcriptional states corresponding to targeted metabolic activation, integrated homeostatic maintenance, and stress-dominated repression, respectively (**Figure 9A**). Similar studies have shown that such distinct functional tendencies represent biologically meaningful equilibrium configurations rather than stochastic variation (Franzosa et al., 2014; Hultman et al., 2015; Vandenkoornhuyse et al., 2015; Trivedi et al., 2020). Importantly, these patterns may be interpreted as alternative system-level responses to drought, where microbial activity either supports host metabolism (K1), maintains function (K2), or undergoes coordinated suppression (KH), highlighting multiple possible adaptive strategies rather than a single optimal response. Importantly, the metatranscriptomic component (**Figure 9**) of this study was designed to characterize functional metabolic activity of the root-associated microbiome rather than its taxonomic composition. This function-centered perspective reflects the principle of microbial functional redundancy, whereby phylogenetically distinct taxa can converge on similar metabolic outputs under environmental stress. Consequently, transcriptional enrichment patterns are interpreted here as indicators of active biochemical programs operating within the plant–microbiome holobiont, rather than as proxies of community structure. Such an approach provides indirect insight into potential links between microbial metabolic processes and host drought adaptation, while acknowledging that causal relationships cannot be directly inferred from these data alone. These patterns are supported by both pathway-level (**Figures 3**, **4**) and gene-level analyses (**Figures 5**, **6**), indicating consistent multi-scale transcriptional regulation. Future work should prioritize functional validation of key genes (e.g., *PT12, PYL10*) using targeted genetic approaches, alongside isotope-based assays to quantify phosphate fluxes. Expanding to diverse genotypes and field conditions, and integrating spatial transcriptomics with microbiome profiling, will be essential to resolve genotype-specific holobiont responses under drought.

## 5 Conclusion

This study provides evidence that root responses to drought in maize in the presence of F. mosseae are genotype-dependent. Drought-tolerant, drought-sensitive, and hybrid genotypes exhibited distinct transcriptomic and pathway-level patterns, indicating that AMF colonization does not induce a uniform response across genetic backgrounds.

In the K1, drought-tolerant genotype, mycorrhizal colonization was associated with enhanced expression of genes related to metabolism and root development, together with reduced representation of stress-related signaling pathways. In contrast, the K2, drought-sensitive genotype showed broader activation of stress- and metabolism-related pathways, while the KH, hybrid genotype displayed intermediate expression patterns across multiple functional categories.

Metatranscriptomic data further indicated genotype-dependent differences in microbial functional activity associated with drought conditions. These observations suggest that drought responses involve coordinated changes between host transcriptional regulation and microbial activity.

However, this study is based on a limited number of genotypes and controlled experimental conditions, which may constrain the generalizability of the findings. In addition, functional interpretations are based on transcriptomic and metatranscriptomic data without direct physiological or metabolomic validation.

Future studies integrating multi-omics approaches and a broader range of genotypes under field conditions will be important to further clarify the mechanisms underlying AMF-mediated drought responses.

## 6 Limitations of the study

The metatranscriptomic states identified in this study represent functional transcriptional profiles rather than strictly defined ecological states. The enrichment patterns reflect differences in expressed functional potential at the time of sampling and do not necessarily correspond to discrete or stable community configurations in an ecological sense. Furthermore, the observed transcriptional shifts may partially arise from changes in microbial community composition, including taxon turnover or relative abundance shifts, which were not directly quantified in this study. Consequently, the inferred functional differences integrate both transcriptional regulation within taxa and potential compositional effects at the community level. Given the limited biological replication and the inherently high variability of root-associated microbial metatranscriptomic datasets, the microbial functional analyses should be considered exploratory and hypothesis-generating rather than definitive taxon-resolved conclusions.

## Data Availability

The datasets presented in this study can be found in online repositories. The names of the repository/repositories and accession number(s) can be found below: Mendeley Data, V2, doi: 10.17632/39byfys8c3.2 The dataset includes (i) total root RNA-seq libraries for host transcriptome profiling (RNAreads.fastq.gz) and (ii) host-depleted meta-transcriptomic libraries enriched for fungal and microbiome transcripts (Unmapped.fastq.gz). (iii) Transcript abundancies are available in ‘Root data’ and ‘Root associated meta-transcriptome data’ folders.
