# Supplementary material for: Funneliformis mosseae enhances drought tolerance in maize inbred lines through root transcriptomic reprogramming
Source: Front Plant Sci. 2026 Jun 18;17:1808527. doi: 10.3389/fpls.2026.1808527 (PMC13323255; doi:10.3389/fpls.2026.1808527)
Supplement: Supplementary file 2 [file Table1.docx]

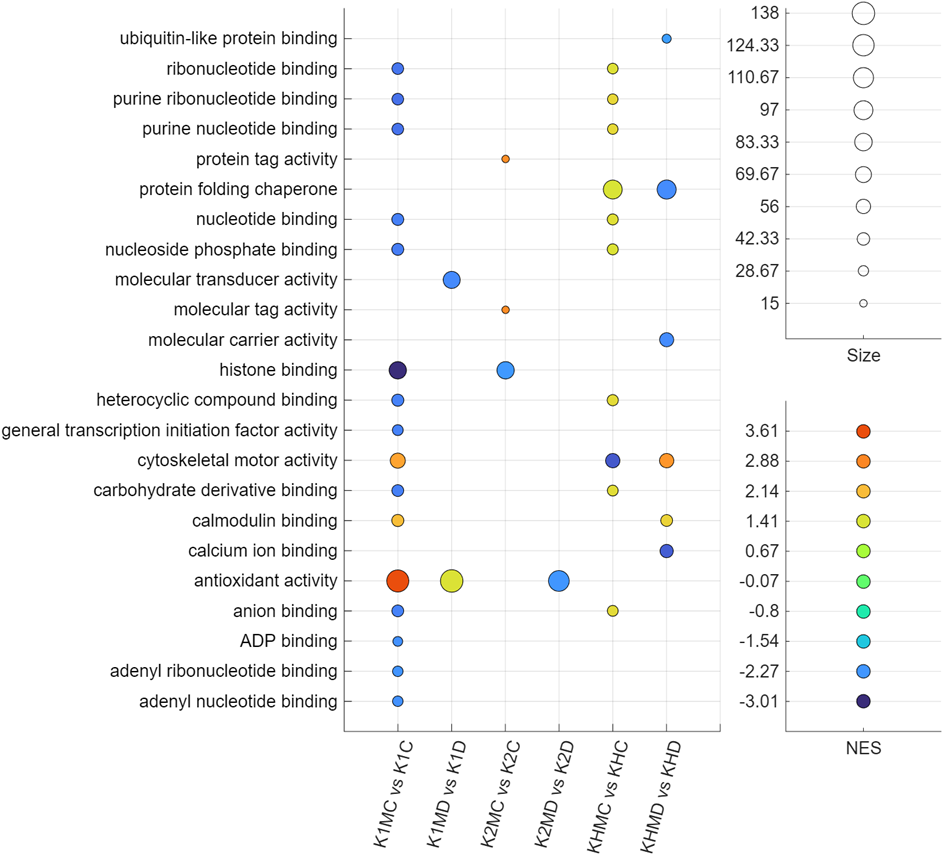


**Supplementary Figure 1. GO molecular function enrichment across treatment contrasts.**

Dot plot showing significantly enriched molecular function Gene Ontology categories among differentially expressed genes in the indicated contrasts (K1MC vs K1C, K1MD vs K1D, K2MC vs K2C, K2MD vs K2D, KHMC vs KHC, KHMD vs KHD).
Dot size represents gene count, and color indicates the normalized enrichment score (NES). Positive NES values denote enrichment, whereas negative values indicate relative depletion.


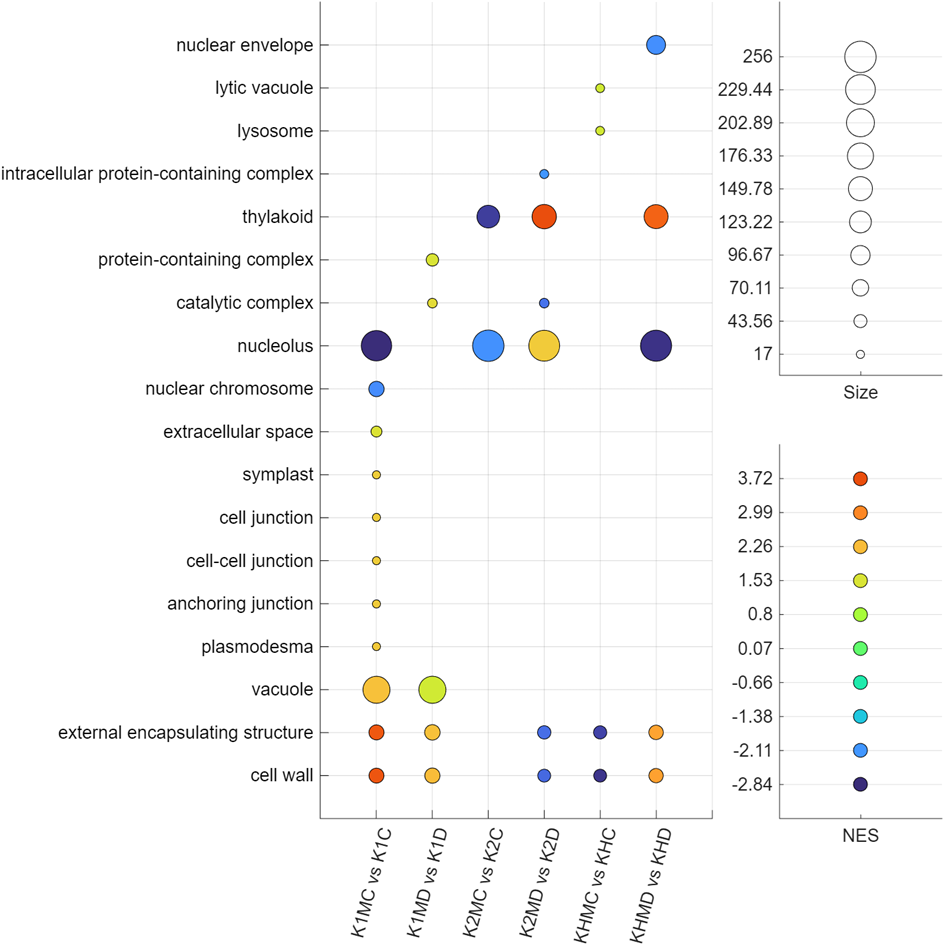


**Supplementary** Figure 2. GO cellular component enrichment across treatment contrasts.

Dot plot displaying significantly enriched cellular component Gene Ontology categories among differentially expressed genes in the indicated contrasts (K1MC vs K1C, K1MD vs K1D, K2MC vs K2C, K2MD vs K2D, KHMC vs KHC, KHMD vs KHD). Dot size corresponds to the number of genes, and color represents the normalized enrichment score (NES), with positive and negative values indicating enrichment and depletion, respectively.
